# Spine extra-osseous chordoma mimicking neurogenic tumors: report of three cases and review of the literatures

**DOI:** 10.1186/s12957-016-0951-0

**Published:** 2016-08-04

**Authors:** Jian Yang, Xinghai Yang, Wujun Miao, Qi Jia, Wei Wan, Tong Meng, Zhipeng Wu, Xiaopan Cai, Dianwen Song, Jianru Xiao

**Affiliations:** Department of Bone Tumor Surgery, Changzheng Hospital, Second Military Medical University, 415 Fengyang Road, Shanghai, China

**Keywords:** Spine extra-osseous chordoma, Neurogenic tumor, Differential diagnosis, Prognosis

## Abstract

**Background:**

For a long time, chordoma has been known as an osseous tumor mainly found at the clivus and sacrococcygeal region. However, spine extra-osseous chordoma (SEC) with a better prognosis than the classic type has been neglected. According to our literature review, only several case reports have been published in English literatures. Here in this article, three cases of SEC, plus a literature review, are presented.

**Case presentation:**

Three cases of SEC were presented from our center. Surprisingly, neurologic tumors were considered as the first diagnosis. Thereafter, en bloc resection was performed in all the three cases. Especially, the dumbbell-shaped one in the cervical spine was removed by en bloc through the combined anterior and posterior approach for the first time. Follow-up within 12–58 months after surgeries proved no recurrence or metastasis.

**Conclusions:**

Spine extra-osseous chordoma, commonly located in the cervical and epidural region, is extremely rarely met. SEC is characterized with less aggressiveness, the lower rate of recurrence and metastasis, and better prognosis than those of the osseous origin. Though complete excision can be achieved generally, differential diagnosis of spine neurogenic tumors and the following en bloc resection should be made as carefully as possible.

## Background

Chordoma is a type of relatively rare tumor with an incidence of about 0.8 per million [1], characterized with bony involvement, strong local invasion, and high recurrence rate. According to the statistics, about 50 % of spine chordomas are found in the sacrococcygeal region, 35–40 % at the clivus, and the rest 10–15 % in the mobile spine (mainly in the cervical region) [2, 3]. Due to its strongly local aggressiveness, chordoma usually comes with a high recurrence rate and a poor prognosis. Carpentier et al. [4] thought that the most significant factor affecting prognosis is the first excision (partial, total, or en bloc resection). Tong et al. [5] found that total spondylectomy was an independent prognostic factor for both local relapse-free survival (LRFS) and overall survival (OS). In the face of this tumor, surgeons are trying their best to accomplish total en bloc spondylectomy even at the cost of neurological function loss. Nevertheless, on account of the specialty and complexity of spine anatomies, as well as the aggressiveness of chordoma, the reported recurrence rate of chordoma is still as high as 17 to 35 %. With the possibility of metastasis, poor prognosis is natural.

Rarely, some chordomas of the spine present no bone involvement, a condition that we call spine extra-osseous chordoma (SEC). SEC, for a long time, has been neglected because of its rather low incidence. Referring to the literatures published in English, 17 cases [6–21] of SEC reported before were classified into two groups: epidural (intraspinal or intraspinal-extraspinal) and intradural. For its rarity and similarity to neurogenic tumors, the majority of these cases might be misdiagnosed as neurinoma before operation. Fortunately, SECs have shown a more favorable prognosis when the en bloc resection was performed. However, if treated as neurogenic tumors, especially for those dumbbell-shaped tumors not wholly resected, the result may be a tragedy. Here, we report three cases of SEC from our center, one of which is the first dumbbell-shaped chordoma resected in en bloc way with a combined posterior-anterior approach up to now, and a systematic review of SEC was performed.

## Case presentation

From 1991 to 2014, 251 spine chordoma surgeries were conducted in our spine tumor center, only three cases being SEC (Table 1). The first case was about a 50-year-old woman with numbness in her right upper limb for 8 years. The cervical MR and CT revealed an intraspinal mass without bone involvement that was soon considered as a neurinoma radiographically. The second patient complained of numbness and pain in the right upper limb and weakness of the lower limbs. A dumbbell-shaped mass with little secondary bone destruction was observed by both MR and CT. The last one came with repetitionary sacrococcygeal discomfort for 17 years and exacerbation for 1 month. With sacrum MR and CT, the dumbbell-shaped neurogenic tumor was suspected. For all of them, chordoma was not the first diagnosis that came into our consideration.

**Table 1:** Summary of SECs reported in the literatures

Case	Sex/age	Systems	Location	MRI	Resection	Capsule	A/T	F/R
1 [8]	M, 69	Pain of left hand and fingers	C (IE)	T1 hyper	TR	ND	ND	ND
2 [7]	M, 38	Neck and shoulder pain	C (IE)	T2 hyper	TR	+	−	84/−
3 [7]	F, 44	Neck and arm pain	C (IE)	T1 hypo	TR	+	−	12/−
4^a^	F, 50	Right upper limb numbness	C (ID)	T1 iso, T2 hyper, enhancement	TR	+	−	12/−
5 [21]	F, 50	Neck pain, right arm numbness	C (ID)	Enhancement	Subtot	−	ND	ND
6 [6]	F, 29	Neuralgia, dysesthesia	C (IE)	T1 hypo, T2 hyper	TR	+	−	18/−
7 [12]	M, 53	Right lower limb weakness, spine paresthesias	C (ED)	T1 hypo, T2 hyper	TR	+	Radio	60/−
8 [16]	M, 5	Neck weakness, stiffness	C (IE)	T1 iso, T2 hyper, enhancement	TR	+	ND	ND
9^a^	F, 67	Right upper limb pain, lower limbs weakness	C (IE)	Enhancement	TR	+	−	13/−
10 [11]	F, 37	Paresthesia of arms	C (ID)	Enhancement	PR	+	−	18/−
11 [18]	M, 65	Gait disturbance	C (ID)	T1 iso, enhancement	PR	+	−	18/−
12 [14]	M, 36	Paraparesis, constipation urinary retention	C (ID) T-L(IM)	T2 hyper, enhancement	partial	ND	Radio	0.75/−
13 [9]	F, 33	Shoulders pain, gait disturbance	T (ID)	ND	TR	ND	ND	12/−
14 [10]	F, 31	Gait disturbance	T (IE)	T1 iso, T2 hypo, enhancement	Partial	ND	Radio	36/−
15 [15]	M, 6	Dorso-lumbar rigidity, buttock pain,	L (IE)	T1/2 iso, no enhancement	ND	+	−	−/−
16 [13]	F, 17	Lower limbs pain	L (ED)	Enhancement	TR	+	−	48/−
17 [19]	F, 28	Low-back, lower limbs pain	L-S (ID)	T1 hyper, T2 iso	TR	+	−	5/−
18 [20]	F, 38	Bilateral buttock pain	S (ID)	T1 iso, T2 hyper	TR	+	ND	6/−
19 [17]	F, 50	Back pain, headache, right leg numbness	C-S (ID/IM)	ND	subtot	+	−	19/−
20^a^	F, 36	Sacrococcygeal region discomfort	S (IE)	Clear margin, enhancement	TR	+	−	58/−

*A/T* adjuvant therapy, *F/R* follow up (month)/recurrence, *hypo* hypointense, *hyper* hyperintense, *iso* isointense, *R* resection, *TR* total resection, *PR* piecemeal resection, *ND* not described, *W* week, *Radio* radiotherapy, *ID* intradural, *ED* epidural, *IE* intraspinal-extraspinal, *IM* intramedullary

^a^Cases presented by our center

All these three patients were treated with surgery of total resection. Pathologically, chordoma was diagnosed in three cases, and all of them recovered completely with no recurrence and distant metastasis within a follow-up ranging from 12 to 58 months. The total en bloc resection ensured the satisfied prognosis.

### Case illustration

**Fig. 1:**
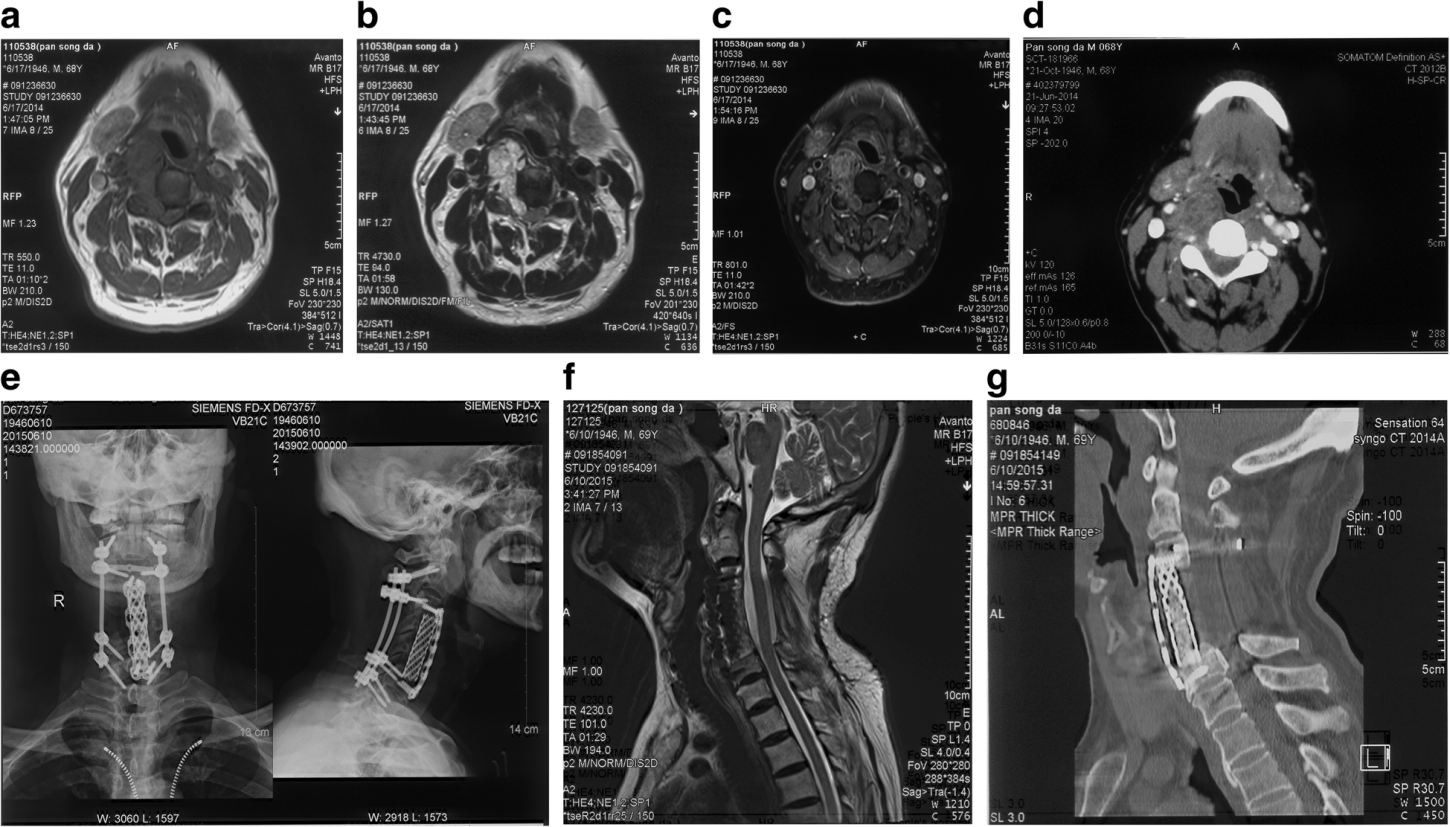
The preoperative and postoperative images. **a**, **b** A dumbbell-shaped and capsuled tumor was showed on T1 and T2, respectively. **c** Slight enhancement after injection of gadolinium. **d** Very little secondary involvement of the bone. **e**, **f**, **g** Postoperative X-ray, MRI, and CT

A 67-year-old man visited our center in 2014, complaining of a 3-month discomfort in the right shoulder, followed by numbness and pain in the right upper extremity and weakness in the lower extremities. Through physical examination, his motor strength of the lower limbs was 4/5, and both the Hoffmann and Babinski reflexes were negative. The cervical magnetic resonance imaging (MRI) demonstrated that a giant dumbbell-shaped mass was located in the right intervertebral foramen of C4 and C5, expanding from C4 to C5 vertebra, with rare involvement of the bone. Inevitably, the spinal cord was compressed. Moreover, hypointense signals on T1 and hyperintense signals on T2 were witnessed (Fig. 1a, b), as well as the heterogeneous enhancement after injection of gadolinium (Fig. 1c). To evaluate the function of vertebral arteries and corresponding risks during the operation, both vertebral artery computed tomography angiography (CTA) and preoperative occlusion test of the right vertebral artery (VA) were performed in our center, revealing the compressed right vertebra artery (Fig. 1d) and the need to rid it. Either MRI or CTA displayed a dumbbell-shaped tumor. A neurogenic tumor was taken into account when he attended the local hospital, of which we doubted to some extent.

To make the preliminary diagnosis of this special mass and a better treatment plan, we first carried out the needle biopsy. The pathological result surprised us that a chordoma existed. In the light of this condition, it was extraordinary for a dumbbell-shaped mass to be diagnosed as chordoma. We believe that it may represent an uncommon morphology of chordoma: spine extra-osseous chordoma (SEC).

### Operation technique

Justified with these preoperative experiences, a posterior-anterior approach was projected for the total en bloc resection of this tumor. Eight lateral mass screws were placed in the lateral masses of C2–3 and C6–7, followed by the left rod fixation and the osteotomy of spinous processes at C4–5. Subsequently, the intraspinal (epidural) capsuled part was dissected from the compressed dura matter but without removal. The involved right fifth nerve root was ligated. The posterior approach was done after fixation and suture.

Then, the patient was placed in the supine position for the anterior approach. When exposing the surface of C3–6 vertebras, a big capsulated mass came into sight. The right vertebral artery was embraced. Taking the aggressiveness and high recurrence rates of chordoma into consideration, ligation of the right VA was performed. After that, with a sagittal excision of C4–5 vertebras, total resection of the tumor was finished successfully, with an appropriate titanium mesh filled with allogeneic bone and a suitable titanium plate placed at C3 and C6 vertebra. The dumbbell-shaped tumor was totally resected without intraoperative complications.

### Pathology

**Fig. 2:**
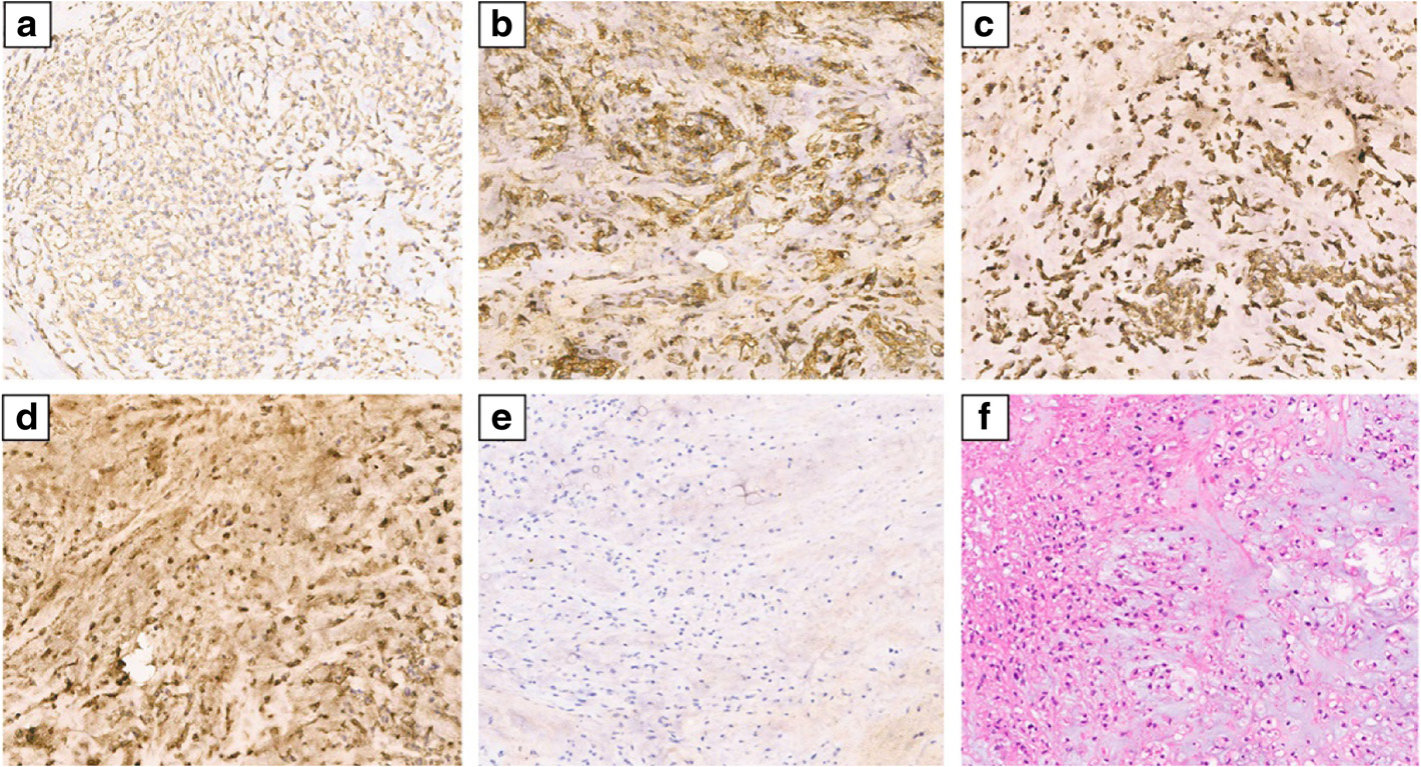
Immunohistochemical reactions. **a** CK pan (+). **b** EMA (+). **c** Vimentin (+). **d** S-100 (+). **e**: Ki67 (5 %); **f** HE staining

Normally, the resected mass was well-capsulated. Microscopically, the mass was composed of lobules full of vacuolated physaliphorous cells rich in intra-cytoplasmic mucin. There was no evidence of mitotic activity. The immunohistochemical result was positive for the existence of S-100, epithelial membrane antigen (EMA), CK (pan), and vimentin. The Ki-67 index was about 5 % (Fig. 2a–e). According to the histological and immunological features, chordoma was diagnosed, conforming to that got by the needle biopsy pathology.

### Follow-up

The patient was able to walk around with the help of a collar 3 weeks after the operation. His discomfort, numbness, pain, and weakness of the lower extremities dwindled soon after the operation, without any perioperative complication. To examine the surgical efficiency, we advised him to take radiotherapy. The cervical CT and MRI at 12 months after the surgery demonstrated no recurrence of the tumor and the perfect fusion of the bone graft (Fig. 1e–g). Further follow-up continues.

### Systematic review

We carried out the literature research by retrieving the keywords like intradural, intraspinal-extraspinal, intraspinal, extra-osseous, and dumbbell-shaped chordoma. What is more, the abstracts were scanned by two authors to choose the suitable articles, and then they read the contents and the references carefully for other possible reports. Sixteen articles containing 17 SEC cases (Table 1) were found. Surprisingly, the occurrence rate of SEC in all chordoma victims was not reported. Thus, we calculated the data in our single center. Two hundred fifty-one spine chordoma surgeries were conducted from 1991 to 2014, and only three cases were about SEC. Then, the occurrence rate of SEC in all chordoma victims was calculated to be 1.195 % (3/251). It is the only occurrence rate of SEC up to now.

As presented in Table 1, there were 11 females and 9 males presenting no significant difference in sex. The average age was 39.1 (ranging from 5 to 69) years old. The clinical symptoms, mainly due to the compression of nerve root and spinal cord, varied from each other. Tumors were mainly located in the cervical region (55 %) and epidural region (intraspinal or intraspinal-extraspinal, 55 %). All cases except two (case 12, 19) occurred as single lesion. In treating these 18 patients, 14 received complete total resection, and 4 received subtotal resection through posterior or post-lateral approach. In terms of adjuvant therapy, only 3 of them received radiotherapy for the incomplete resection. The recorded postoperative follow-up lasted for 3 weeks to 84 months, and only one (case 12) died of respiratory embarrassment, diaphragmatic paralysis, and quadriparesis caused by cervical compression 3 weeks after thoracic operation. Recurrence or metastasis occurred in just one of them (case 19) due to the subtotal or partial removal.

Microscopically, they presented the classic features of chordomas such as vacuolated physaliphorous cells embedded in lakes of extracellular mucin. Notably, the status of mitotic activity in five patients was respectively described as: 5 %, minimal, low, scanty, and no. This reminds us that SEC may be characterized with lower aggressive behavior. Immunohistochemically, positive immunoreactions were observed for the appearance of cytokeratin, epithelial membrane antigen (EMA), vimentin, and S-100. With antibodies against carcinoembryonic antigen (CEA), aktin, and desmin, negative reactions were observed. Ki67, a proliferation index, was recorded only in two cases (about 1 %).

### Discussion

Chordoma, comprising 1 to 4 % of all primary bone malignancy, is a type of locally aggressive tumor predominantly involving the sacrococcygeal and the clival region. Only 10–15 % of the chordomas are located in the mobile spine, in particular, the cervical region. Histologically, chordomas are classified into conventional, chondroid, and dedifferentiated variants. The chordoma is well-known for its slow-growing but strong local aggressiveness and high recurrence rate. At present, therapeutic options mainly include surgery, radiotherapy, and targeted therapy. Chemotherapy was seldom used. Surgery, especially the total en bloc spondylectomy, is thought to be the most preferred treatment for chordoma even at the cost of neurological function [4, 5]. However, the surgery is not able to ensure the absolute survival. Given the anatomical complexity of the spine, it is a big challenge for surgeons to resect the tumor totally. What is more, the poor response to traditional radiotherapy, especially chemotherapy, also brings down the treatment’s effect. Due to its aggressiveness, recurrence, and the opportunity of metastasis, chordoma often has a terrible prognosis with the 5- and 10-year overall survival (OS) rate of 65–78.6 % and 30–39.9 %, respectively [1, 9, 22–24].

Chordoma is characterized with bone involvement. Yet, the 20 SEC cases showed intradural or epidural (intraspinal-extraspinal) or minimal secondary bone involvement. As was illustrated by MR, 8 of the 20 cases presented hypointense to isointense signals on T1-weighted image, and 6 showed hyperintense signals on T2-weighted image. Furthermore, 9 of them showed enhancement after the injection of gadolinium. These features were similar to those of neurinoma. Before the operation, 6 cases were first misdiagnosed with neurinoma. Others misdiagnosis included arachnoid cyst, meningioma, and myxopapillary ependymoma. Therefore, for their special locations (intradural or intraspinal-extraspinal), shapes and similar MR manifestations, the radiographic differential diagnosis between SECs and neurogenic tumors becomes quite difficult. Despite this, differential diagnosis between them must be taken into consideration in the face of possible neurogenic tumors.

Histologically, the main body of classic chordomas contains normal physaliphorous cells and lobulated structures. In the cases collected in our study, most of the histological and immunohistochemical features were consistent with those of the classic chordoma. Positive immunoreaction was strong for the appearance of cytokeratin, epithelial membrane antigen (EMA), vimentin, and S-100. Other positive immunohistochemical findings included the platelet-derived growth factor receptor (PDGFR) α and β, Ki67. Yet, antibodies against CEA, aktin, and desmin did not show up. Few studies [25–27] reported that MIB-1, an antibody reactive to Ki67 protein, ranged from 0 to 16.2 %, and was statistically associated with chordoma’s local recurrence. For the present SECs, Ki67 was recorded only in two cases, with a relatively low level of 1 %. Similarly, the mitotic activity of the several reported cases was significantly low. These indicated that SEC may have a less aggressive biological behavior. Brachyury, a newly found biomarker in chordoma, has been widely studied and used to diagnose chordoma [28, 29]. Though seldom used in the present cases, it can be employed more frequently for the diagnosis and differential diagnosis of SEC as well.

The postoperative recurrence, approximately 17 to 35 %, is thought to be a very important predictive prognosis indicator for classic chordomas. Additionally, classic chordoma has the potentiality to metastasize to the lung, bone, and liver in about 3 to 48 % patients [5, 23, 30–32]. Among them, the chordoma is most likely to metastasize to the lungs (58 %), then to the lymph nodes (33 %), to the liver (22 %), to the bone (17 %), and to the skeletal muscle (9 %) [33]. In contrast, recurrence and metastasis appeared in only one SEC, though a total of four subtotal resections were performed. The most important reason is that the sharply circumscribed margins and the absence of bone involvement made complete resection possible. Another reason may be the lessened aggressiveness of SEC. However, the 19th patient suffered from a serious recurrence and metastasis after the subtotal resection of intramedullary tumor. Hence, total en bloc resection should also be suggested to avoid recurrence and metastasis for SECs, especially for the dumbbell-shaped ones. Alotaibi et al. [34] also reviewed the patients suffering intradural chordoma at the skull base with a good result of 5-year recurrence-free survival (RFS) and overall survival (OS), particularly the patients who had undergone complete resection. Without any doubt, the low recurrence and metastasis rates contribute to the better prognosis than the classic.

In summary, SEC is an extremely rare and special subtype of chordoma with the following characteristics: (1) the absence of bone involvement; (2) the similar morphology with neurogenic tumors; (3) the low aggressive biologic behavior; (4) a better prognosis than the classic chordoma. Because of the limited quantities, further studies are required to better understand SEC’s origination, immunochemistry, differential diagnosis, biologic behavior, and prognosis.

## Conclusions

Spine extra-osseous chordoma, usually located in the cervical and epidural region, is extremely rare. SEC is characterized with the less aggressiveness, the lower rate of recurrence and metastasis, and the better prognosis than those of the osseous origin. Though complete excision can be achieved generally, differential diagnosis of spine neurogenic tumors and the following en bloc resection should be made as carefully as possible.

## Abbreviations

CEA: Carcinoembryonic antigen
CK: Cytokeratin
CT: Computed tomography
EMA: Epithelial membrane antigen
LRFS: Local relapse-free survival
MRI: Magnetic resonance imaging
OS: Overall survival
PDGFR: Platelet-derived growth factor receptor
SEC: Spine extra-osseous chordoma
